# Neuronal architecture of the second-order CO_2_ pathway in the brain of a noctuid moth

**DOI:** 10.1038/s41598-020-76918-1

**Published:** 2020-11-16

**Authors:** X. Chu, P. KC, E. Ian, P. Kvello, Y. Liu, G. R. Wang, B. G. Berg

**Affiliations:** 1 Chemosensory Laboratory, Department of Psychology, Norwegian University of Science and Technology, NTNU, Trondheim, Norway; 2 Department of Teachers Education, Norwegian University of Science and Technology, NTNU, Trondheim, Norway; 3 State Key Laboratory for Biology of Plant Diseases and Insect Pests, Institute of Plant Protection, Chinese Academy of Agricultural Sciences, Beijing, China

**Keywords:** Neuroscience, Systems biology

## Abstract

Many insects possess the ability to detect fine fluctuations in the environmental CO_2_ concentration. In herbivorous species, plant-emitted CO_2_, in combination with other sensory cues, affect many behaviors including foraging and oviposition. In contrast to the comprehensive knowledge obtained on the insect olfactory pathway in recent years, we still know little about the central CO_2_ system. By utilizing intracellular labeling and mass staining, we report the neuroanatomy of projection neurons connected with the CO_2_ sensitive antennal-lobe glomerulus, the labial pit organ glomerulus (LPOG), in the noctuid moth, *Helicoverpa armigera.* We identified 15 individual LPOG projection neurons passing along different tracts. Most of these uniglomerular neurons terminated in the lateral horn, a previously well-described target area of plant-odor projection neurons originating from the numerous ordinary antennal-lobe glomeruli. The other higher-order processing area for odor information, the calyces, on the other hand, was weakly innervated by the LPOG neurons. The overlapping LPOG terminals in the lateral horn, which is considered important for innate behavior in insects, suggests the biological importance of integrating the CO_2_ input with plant odor information while the weak innervation of the calyces indicates the insignificance of this ubiquitous cue for learning mechanisms.

## Introduction

Insects navigate through a complex environment by using multisensory information. Herbivorous species detect variations in plant-emitted carbon dioxide (CO_2_) concentration for the purpose of determining the host plant quality. Actually, small fluctuations of atmospheric CO_2_ may affect many behaviors in insects, from foraging to oviposition, reviewed by Stange and Stowe^1^, Guerenstein and Hildebrand^2^, and Cummins et al.^3^. In the polyphagous, lepidopterous *Helicoverpa armigera,* for instance, CO_2_ signals ensure that the larvae get access to the metabolically most active parts of the plant during feeding^4^. Furthermore, adult hawkmoths of the species *Manduca sexta* detect freshly opened and nutritious flowers of *Datura wrightii* based on their elevated CO_2_ emission^5^. Another example is the female cactus moth, *Cactoblastis cactorum,* utilizing ambient CO_2_ levels to locate the photosynthetically most active part of the host plant cactus, *Opuntia* *stricta,* for egg laying^6^*.*

Previous studies on different lepidopteran species have shown that external CO_2_ fluctuations are detected by sensory neurons housed in a specialized organ located on a section of the mouthparts called the labial palps^6–8^. In *H. armigera*, the labial pit organ (LPO) houses ca. 1200 CO_2_ sensory neurons^9^. These LPO neurons express three types of gustatory receptor genes^10,11^. Previous mass staining experiments from the relevant segment of the labial palps have demonstrated sensory fibers targeting various sites including bilateral projections to one glomerulus in each antennal lobe (AL), called the LPO glomerulus (LPOG)^9,12^. A recent study reported that the LPO sensory projections which include bilateral, ipsilateral, and contralateral neurons, target the LPOG exclusively^13^.The remaining AL glomeruli, comprising three male-specific glomeruli (the macroglomerular complex, MGC) and ca. 75 ordinary glomeruli, receive input from antennal neurons tuned to pheromones and plant odors, respectively^14^. None of these sensory neurons, which form the antennal nerve, target the LPOG.

**Figure 1:**
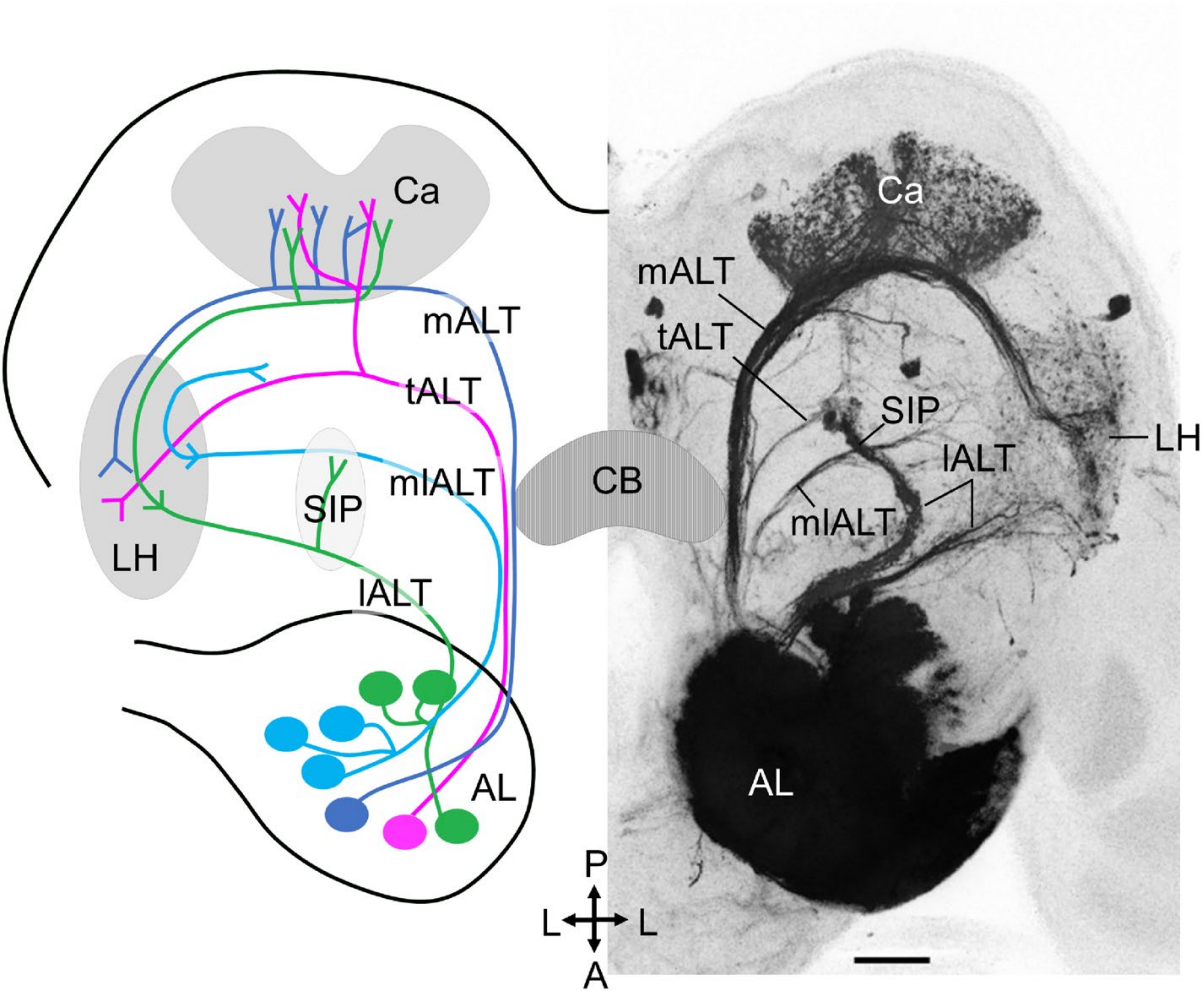
Overview of antennal-lobe tracts (ALTs) in moth. *Left*: Schematic drawing of the medial, transverse, mediolateral, and lateral ALT (mALT, tALT, mlALT, and lALT). *Right*: A corresponding confocal image obtained by mass filling the AL. Ca, Calyces of the mushroom body; LH, lateral horn; SIP, superior intermediate protocerebrum; CB, central body. A, anterior; L, lateral; P, posterior; Scale bar, 50 μm.

In the AL, all sensory neurons including those projecting both from the antenna and the LPO, make synapses with second-order neurons within the numerous glomeruli. There are two types of AL neurons, local interneurons (LNs) and projection neurons (PNs). The latter cells carry odor information to higher processing centers in the protocerebrum via several parallel antennal-lobe tracts (ALTs; Fig. 1). The most prominent tract, the medial ALT, is reported to connect the antennal lobe with the calyces of the mushroom bodies (Ca, center for associative learning)^15,16^ and the lateral horn (LH, center for innate behavior)^17^ in many moths, including *M. sexta* ^18^, *Bombyx mori*^19,20^, *Heliothis virescens* ^21,22^, *Helicoverpa zea*^23^, *Helicoverpa assulta*^24^, and *H. armigera*^25^. Two other well-described tracts are the lateral and mediolateral ALT, both of which target protocerebral regions mainly outside the Ca^18,26^.

In moth, there are three minor tracts formed by relatively few neurons, two of which are the transverse ALT^27^ and the dorsomedial ALT^18,27^. The transverse tract projects partly in parallel with the mediolateral tract. The mediolateral tract splits off from the medial tract at the anterior edge of the central body (CB), while the transverse tract splits off at the posterior edge of the CB (Fig. 1). The number of axons forming the transverse tract is slightly lower than that in the mediolateral tract (in *Drosophila melanogaster*, 60 axons in the transverse tract vs. 80–100 axons in mediolateral tract)^28^. The transverse tract was first described in *D. melanogaster*^28^. Lately, individual neurons confined to this tract have been described in various insect species, such as *H. virescens*^27,29^, *H. armigera*^26^, *Hieroglyphus banian*^30^, and *D. melanogaster*^31,32^. The other minor tract, the dorsomedial tract, consisting of a few neurons only, was first reported in *M. sexta*^18^. So far, only two studies have described the complete morphology of individual neurons confined to this tract in the noctuid moth^21,26^.

Generally, output neurons originating from the ordinary glomeruli and the MGC have been relatively well studied in moths, reviewed by Martin et al.^33^. Previous studies in several species have shown that the second-order circuits for pheromone and plant odor signals are mainly separated^18,25^. The projections of LPOG output neurons, on the other hand, have been poorly described. So far, only one study on this system has been carried out, reporting two types of CO_2_-responding neurons originating from the LPOG in *M. sexta*^34^. Based on the previously well-described chemosensory pathway including (1) distinct AL glomeruli for input about pheromones, plant odors, and CO_2_, respectively^9,13,14^, and (2) a general separation of pheromone and plant odor signals at the subsequent synaptic level^18,25,35^, it is particularly interesting to investigate how second-order neurons carrying signals from CO_2_ are arranged. In the present study, we performed a series of intracellular staining and mass staining experiments enabling tracing of the PNs forming this pathway in *H. armigera*. Totally, five morphological LPOG neuron types passing along different ALTs were found. Their projection targets were mainly segregated from pheromone output regions but overlapped to a certain extent with terminals of plant odor neurons. Notably, most LPOG PNs were confined to other tracts than the prominent medial tract.

## Results

### Outline of protocerebral regions serving as targets for LPOG PNs

**Figure 2:**
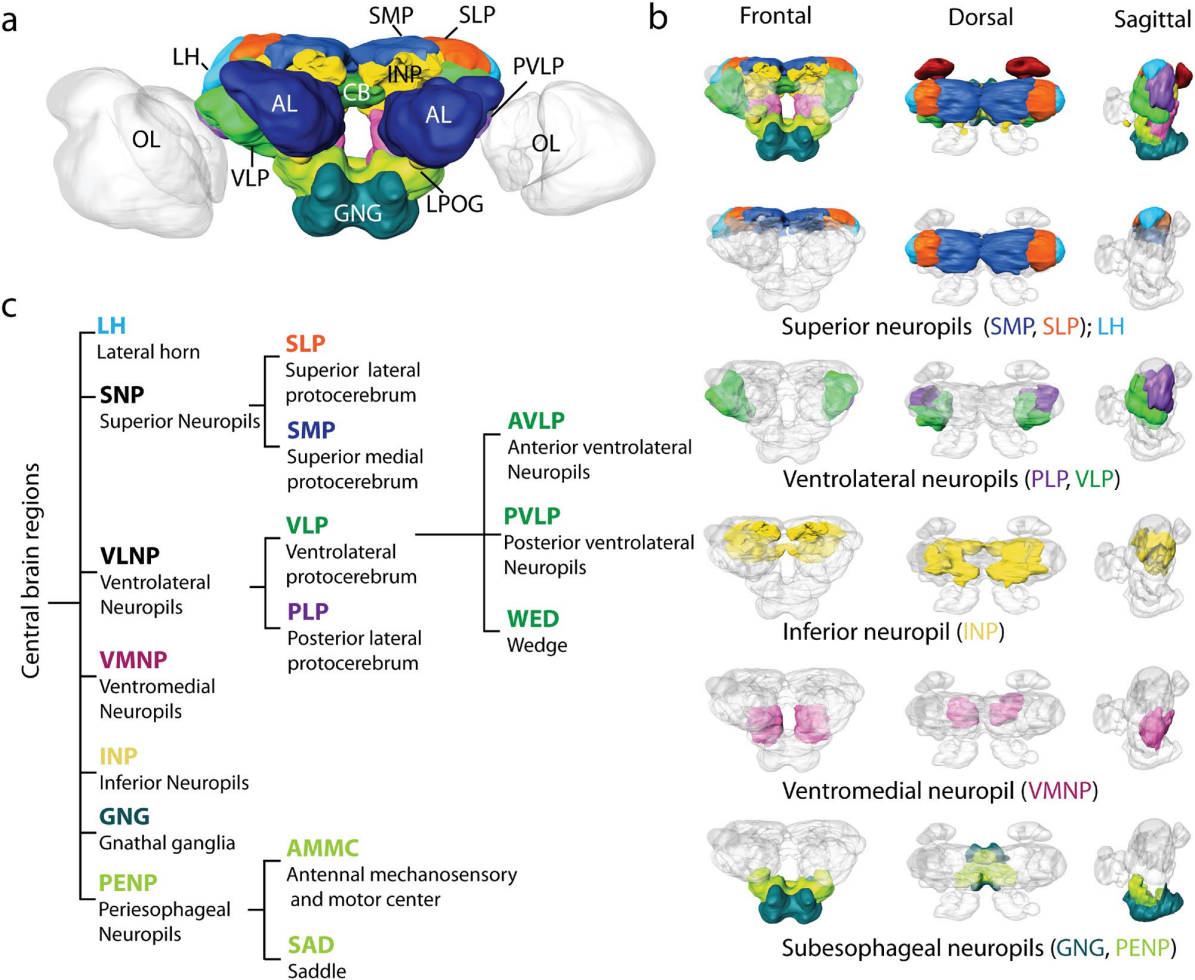
Layout of LPOG PN output regions. (**a**)Three-dimensional surface model of the representative brain of *Helicoverpa armigera*^25^, in frontal view, including the relevant neuropils in. AL, antennal lobe; CB, central body; GNG, gnathal ganglion; INP, inferior neuropils; LH, lateral horn; OL, optical lobes; SMP: superior medial protocerebrum; SLP, superior lateral protocerebrum; VLP ventrolateral protocerebrum. Scale bar: 100 μm. (**b**) The main defined neuropils targeted by LPOG neurons, indicated by distinct colors. From top to bottom: All defined central brain neuropils (*colored*) shown together with the continuous mass of undefined regions (*gray*). Scale bar: 100 μm. (**c**) Hierarchical diagram showing the neuropils in (**b**) including sub-regions.

To provide a framework for describing the main target areas of the LPOG PNs, we reconstructed a selection of protocerebral regions from the confocal image stack previously utilized for creating the representative brain model^25^. In addition to the main neuropil structures originally included in this 3D brain atlas, we added an assembly of nine areas in order to determine the main output regions of the LPOG neurons identified here (Fig. 2a, b). All newly reconstructed regions are listed and presented in Fig. 2c.

### ***The PN pathway carrying CO***_***2***_*** input is distinct from the canonical olfactory PN pathway***

**Figure 3:**
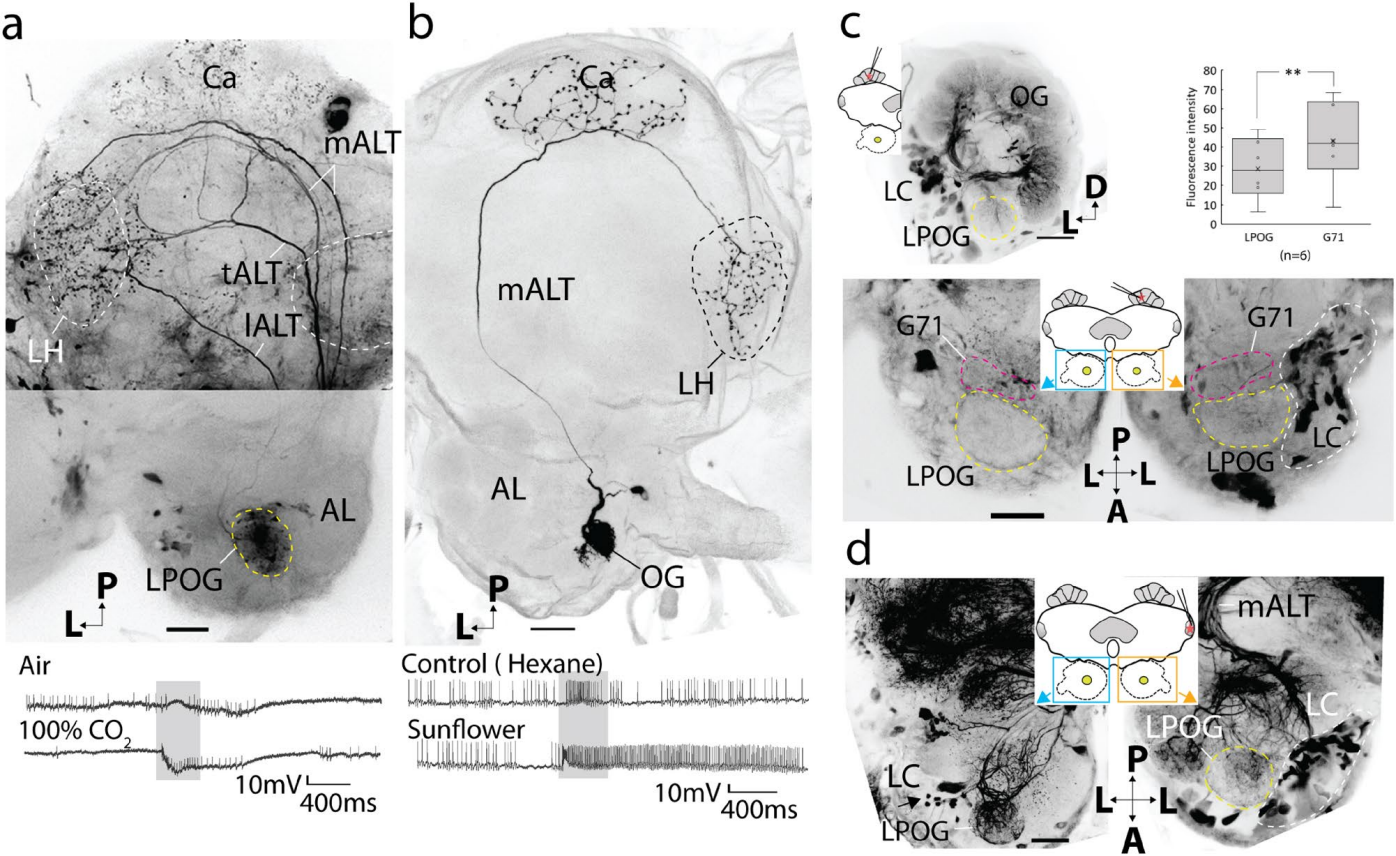
Comparison of LPOG PNs versus PNs connected with ordinary glomeruli. (**a**) Mass staining of LPOG-neurons via anterograde labeling from the LPOG. Labeled neurons confined to the transverse, medial, and lateral ALT (tALT, mALT, and lALT). Overlapping projection terminals occur in the lateral horn (LH) whereas the calyces (Ca) is weakly innervated. The tALT PNs bend off from the mALT at the posterior edge of the central body (dashed *white* line). Spiking activity of the mass stained LPOG neurons during application of fresh air and CO_2_ is shown below. (**b**) Confocal image of a typical plant-odor sensitive mALT neuron with extensive innervations in the Ca. Spiking activity during stimulation with hexane and plant-odor is shown below. (**c**) Retrograde labeling from Ca. The upper confocal image shows substantially weaker labeling in the LPOG than in the ordinary glomeruli in the ipsilateral AL (frontal view). The box plot demonstrates the difference in fluorescence intensity between the LPOG and the neighboring glomerulus, G71. The additional confocal image below, including both ALs in dorsal view, demonstrates the location of the LPOG (*yellow* dashed circle) and G71 (*red* dashed circle). (**d**) Retrograde labeling from LH. Confocal image of the contralateral AL (*left*), showing extensive labeling in the LPOG. Nine stained somata (arrow) in the lateral cell body cluster (LC), connected with the LPOG, indicates at least nine bi- or contralateral LPOG neurons targeting the LH. Scale bars: 50 μm. A, anterior; L, lateral; P, posterior.

In order to obtain an anatomical overview of the PN pathway carrying CO_2_ information in the moth brain, a series of focused mass staining experiments from the LPOG was first performed. Confirmation about dye application into the LPOG, and the LPOG exclusively, was obtained by examining the presence of labeled sensory neurons in the LPO (see confocal scanning in Supplementary Fig. S1) and a lack of staining in the ventral AL glomeruli, located adjacent to the lateral cell body cluster (LC). As demonstrated in one of the preparations, including the highest number of simultaneously labeled LPOG PNs (Fig. 3a), the projection pattern demonstrated extensive labeling in the LH. This pattern originated from overlapping terminals of axons passing along three distinct ALTs, the transverse, medial, and lateral tract. The transverse-tract neurons were particularly prominent. The relatively uncommon transverse-tract neuron type is previously reported to carry information from a few AL glomeruli only^26^, and our data show that the LPOG is one of them. In addition to targeting the LH, some LPOG output neurons also projected to the Ca, however, with substantially weaker innervations. In order to compare the projection patterns of the LPOG PNs and a typical medial-tract PN, we placed confocal images of the two PN categories next to each other (Fig. 3a, b). The considerably stronger staining of the Ca in the medial-tract PN is obvious. Furthermore, the mass stained preparation in Fig. 1, including AL output neurons originating from seemingly all glomeruli, displays extensive innervations in the Ca as well. To quantify the characteristic difference in the terminal patterns of LPOG PNs versus PNs originating from ordinary glomeruli, we measured the fluorescence intensity within the Ca and the LH region in each of the three stained preparations referred to above. The Ca/LH-ratios were then calculated and found to be 0.42 for the LPOG PNs (Fig. 3a), 1.35 for the AL mass-stained preparation (Fig. 1), and 0.89 for the single medial-tract PN (Fig. 3b). Thus, we assumed that the LPOG PNs were relatively weakly connected to the Ca.

Next, to confirm the contrasting distribution of axon terminals in the LH vs. Ca of LPOG PNs, we carried out two types of retrograde mass staining experiments. We first injected fluorescent dye into the Ca, which resulted in extensive staining of all glomeruli in the ipsilateral AL except for one, the LPOG (Fig. 3c). By quantifying the mean fluorescent intensity in the ipsilateral LPOG and the neighboring glomerulus, G71 (Fig. 3c and Supplementary Fig. S2), we found that the LPOG was consistently weaker stained (*t*(5) = 4.97, *p* = 0.004). This confirmed our hypothesis of a relatively weak connection between the LPOG and the Ca. The second retrograde mass staining experiment involved application of dye into the lateral protocerebrum, including LH. This caused not only labeling of all glomeruli in the ipsilateral AL, including the LPOG, but also uncovered an assembly of stained axons linked to the LPOG in the contralateral AL (Fig. 3d). Here, nine labeled LC somata connected with the innervated glomerulus could be seen—indicating the presence of at least nine bilateral or contralateral LPOG PNs. Altogether, the results from the three kinds of mass staining experiments revealed that the LPOG, receiving input from the labial palp, has an output pathway differing substantially from that of PNs linked to the other AL glomeruli, receiving input from the antennae. The main characteristics of the LPOG pathway include: 1) a prominent involvement of the transverse ALT, 2) relatively weak terminal innervations of the Ca, and 3) a significant proportion of bilateral/contralateral PNs.

### LPOG neurons are uniglomerular PNs with rich morphological diversity

**Figure 4:**
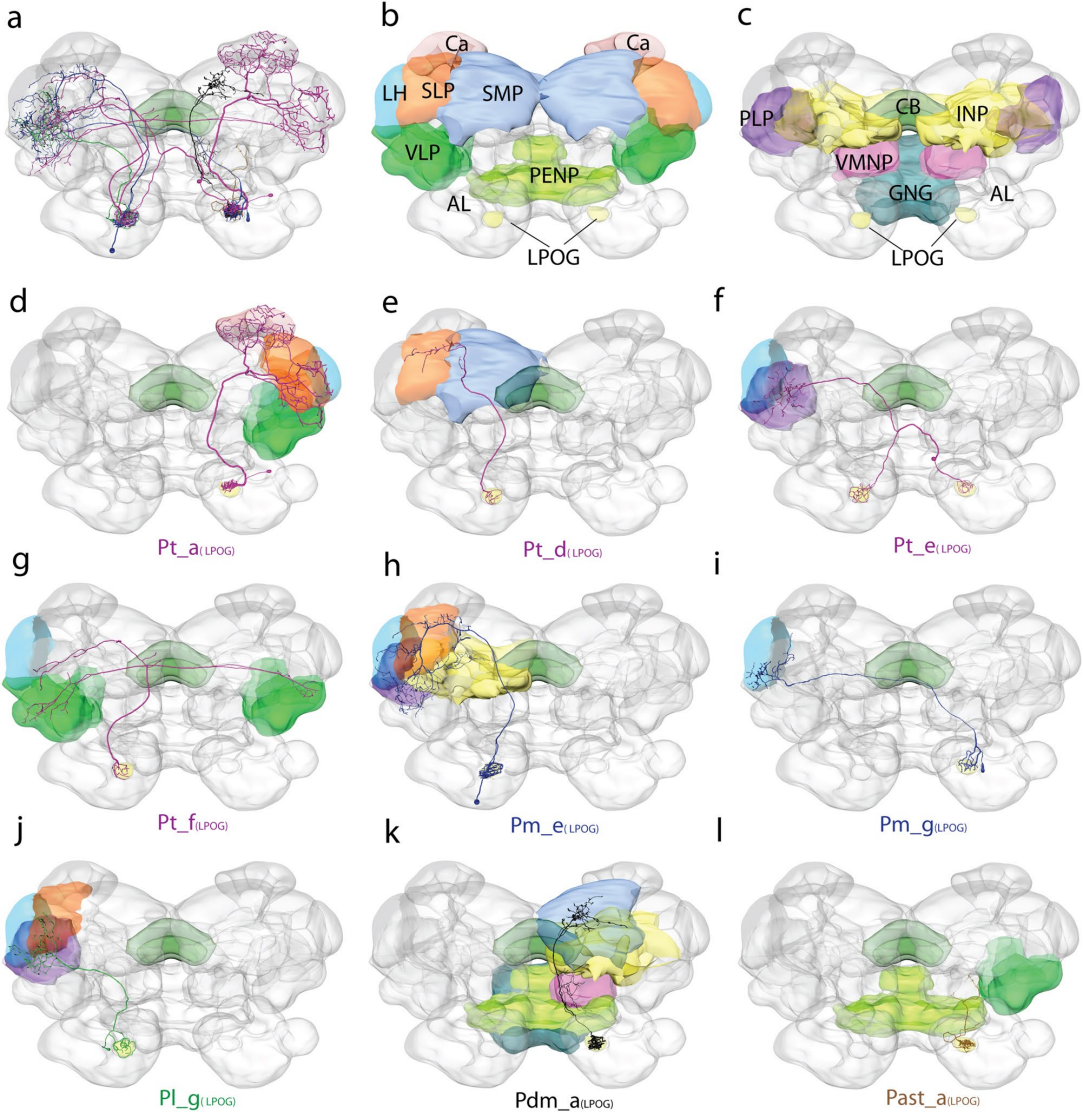
Overview of the anatomical architecture of the LPOG output neurons. (**a**) Summary diagram of the nine LPOG PN subtypes, color-coded according to the tract they are confined to: Transverse-tract neurons in *magenta*, medial-tract neurons in *blue*, lateral-tract neuron in *green*, dorsomedial tract neuron in *black*, and neuron in the antennal subesophageal tract in *orange*. Scale bar: 100 μm. (**b–c**) Summary diagram of the brain neuropils targeted by the LPOG PNs, color-coded in correspondence with all other figure panels. AL, antennal lobe; GNG, gnathal ganglion; INP, inferior neuropils; LH, lateral horn; PENP, periesophageal neuropils; PLP, posterior lateral protocerebrum; SMP, superior medial protocerebrum; SLP, superior lateral protocerebrum; VLP, ventrolateral protocerebrum; VMNP, ventromedial neuropils. (**d–l**) 3D reconstructions of the nine LPOG PN subtypes manually registered into the representative brain (dorsal view).

As the two PN pathways, devoted to CO_2_ and ordinary odorants, respectively, display substantially different projection patterns at the neuron population level, we next aimed at investigating how these differences were expressed at the single-neuron level. We thus carried out intracellular dye injection into the thick dendrites of LPOG PNs. Fifteen LPOG neurons were morphologically identified, all having glomerular dendritic arborizations focused in the LPOG solely. These neurons were confined to five different tracts: ten to the transverse ALT, two to the medial ALT, one to the lateral ALT, one to the dorsomedial ALT, and one to the antenno-suboesophageal tract (AST). Naming of neuron types and subtypes is an adaptation of the system used in previous studies^18,26,27^. This implies that the five neuron types were named according to the tract they projected along (i.e., Pt, Pm, Pl, Pdm, and Past) whereas the neuron subtypes were classified according to their axonal projection patterns in the protocerebrum. For the name, Pt_a_(LPOG)_, “*P”* indicates the PN category, “*t*” applies to the transverse-tract type, “*a”* refers to the PN subtype, while *“*_*(LPOG)*_” represents the uniglomerular dendritic arborization. An overview of the individually stained neurons and their corresponding output regions is shown in Table 1 and Fig. 4.

**Table 1:** Overview of individual LPOG PNs confined to five different tracts.

Tracts	Soma localization	Soma diameter (µm)	Dendritic arborisation (LPOG)	Ipsi-, contra-, or bilateral projections	Output regions	Number	Previous Reports LPOG PNs
***tALT***						*Total:10*	
Pt_a_(LPOG)_	LC	14.60 ± 0.70 (n = 3)	UG	Ipsilateral	Ca, LH, SLP, PLP, VLP	3	PIa(G)^34^
Pt_d_(LPOG)_	–	–	UG	Ipsilateral	SLP, SMP	1	**Novel**
Pt_e_(LPOG)_	MC	10.37 ± 0.62 (n = 3)	UG (LPOG^b^)	Contralateral	LH^c^, PLP^c^	3	Protocerebral neuron^34^
Pt_f_(LPOG)_	LC	8.58 (n = 1)*	UG	Bilateral	LH, VLP^b^	3	**Novel**
***mALT***						*Total :2*	
Pm_e_(LPOG)_	AC	18.10	UG	Ipsilateral	LH, SLP, INP, PLP	1	**Novel**
Pm_g_(LPOG)_	LC	9.98	UG	Contralateral	LH^c^	1	**Novel**
***lALT***							
Pl_g_(LPOG)_	LC	6.27	UG	Ipsilateral	LH, SLP, PLP	1	**Novel**
***dmALT***						*Total:1*	
Pdm_a_(LPOG)_	–	–	UG	Ipsilateral	SMP, INP, VMNP, AMMC, GNG	1	**Novel**
***AST***						*Total:1*	
Past_a_(LPOG)_	GNG	4.53	–	Ipsilateral	AMMC, VLP	1	**Novel**

AC, anterior cell body cluster; ALT, antennal lobe tract; AMMC, antennal mechanosensory and motor center; AST, antennal subesophageal tract; ^b^; bilateral; ^c^, contralateral; Ca, calyx; dmALT, dorsomedial ALT; GNG, gnathal ganglion; INP, inferior neuropil; lALT, lateral ALT; LC, lateral cell body cluster; LH, lateral horn; mALT, medial ALT; MC, medial cell body cluster; PNs, projection neurons; PLP, posterior lateral protocerebrum; SLP, superior lateral protocerebrum; SMP, superior medial protocerebrum; tALT, transverse ALT; UG, uniglomerular; VLP, ventrolateral protocerebrum; VMNP, ventromedial neuropil; *, the soma diameter of one of the three Pt_f_(LPOG)_ PN is stated, the two remaining somata were not visible.

### The main share of stained LPOG neurons passed along the transverse antennal-lobe tract

**Figure 5:**
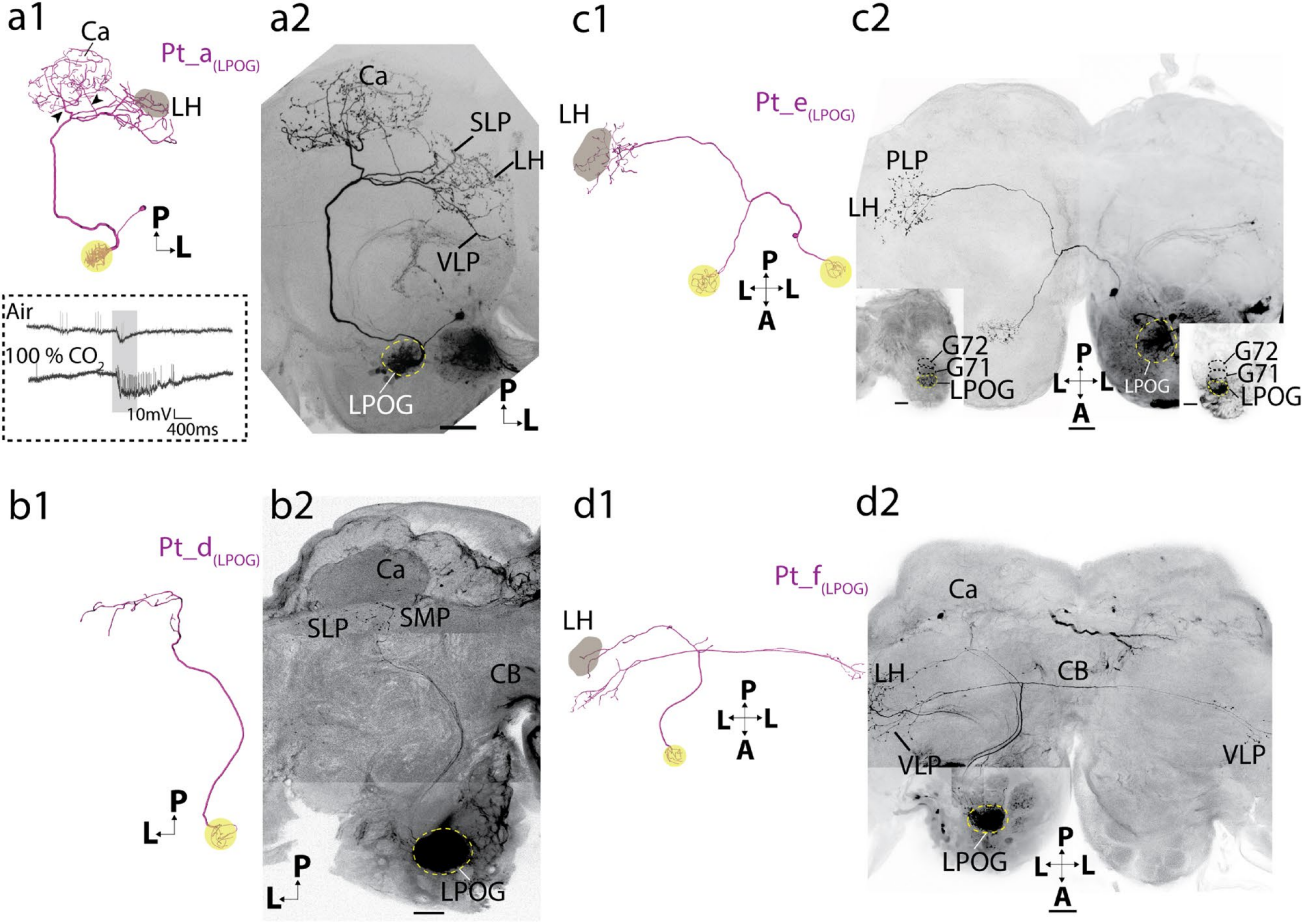
Four transverse-tract LPOG neurons, including two ipsilateral and two bilateral subtypes. (**a1, a2)** 3D reconstruction and confocal image of a Pt_a_(LPOG)_ neuron subtype. The ipsilateral neuron projected to the calyces (Ca) via two axonal fibers (arrowheads) before terminating in the lateral horn (LH), superior lateral protocerebrum (SLP), and ventrolateral protocerebrum (VLP). The soma was in the lateral cell body cluster. (Spike activities during stimulation with fresh air and CO_2_ is shown to exemplify how LPOG neurons were identified during the experiment.) (**b1, b2)** 3D reconstruction and confocal image of a Pt_d_(LPOG)_ neuron subtype. The ipsilateral neuron originated in the LPOG and projected to the superior medial protocerebrum (SMP) and SLP without innervating the Ca. (**c1, c2)** 3D reconstruction and confocal image of a Pt_e_(LPOG)_ neuron subtype. This bilateral neuron arborized in the LPOG of both ALs and terminated in the LH contralateral to the site of the cell body. The two LPOGs were connected via the antennal-lobe commissure. The soma was in the medial cell body cluster. The neuron was co-stained with a weakly labeled lateral-tract LPOG neuron. The two insets in **c2** are confocal images showing the dendritic arborizations in each AL. The LPOG is located adjacent to glomerulus G71. (**d1, d2)** 3D reconstruction and confocal image of a Pt_f_(LPOG)_ neuron subtype. This bilateral LPOG neuron terminated in the ipsilateral LH and in the VLP of both hemispheres. The neuron was co-stained with a Pt_d_(LPOG)_ neuron and a lateral-tract LPOG neuron. (The reconstruction of neuronal dendrites in **b1**, **c1**, and **d1** are shown for illustrative purposes only. Due to prestaining/mass staining of the LPOG, the detailed arborizations were not visible.) All images are in dorsal orientation. CB, central body; A, anterior; L, lateral; P, posterior; Scale bars: 50 μm.

Even though relatively few projection neurons are reported to connect the AL to the protocerebrum via the transverse tract, the main proportion (more than 65%) of labeled neurons originating from the LPOG passed along this route. The transverse-tract neurons were morphologically diverse, including both uni- and bilateral neurons. They all left the AL together with the medial ALT and projected along its path until the posterior edge of the CB. Here, they bent off from the medial tract, passing along the pedunculus before projecting their wide-spread terminal branches in different areas of the ipsilateral and/or contralateral protocerebrum. The transverse-tract neurons comprised four subtypes, two unilateral and two bilateral (Fig. 5).

#### Unilateral transverse-tract neurons

Four of the ten labeled transverse-tract LPOG PNs were unilateral. Three of these unilateral neurons were morphologically similar forming one distinct subtype and the fourth constituted another subtype. The three first-mentioned neurons were the only ones innervating the Ca. In addition, these neurons projected to several regions in the lateral protocerebrum, including the LH, superior lateral protocerebrum (SLP), posterior lateral protocerebrum (PLP), and ventrolateral protocerebrum (VLP, Figs. 4d, 5a). This neuron subtype which was named Pt_a_(LPOG)_, is similar to an LPOG PN previously described in *M. sexta*^34^—by then, defined as a medial-tract PN. The fourth unilateral transverse-tract neuron, constituting a distinct subtype, bypassed the Ca anteriorly and targeted the lateral part of the SLP and superior medial protocerebrum (SMP, Figs. 4e, 5b). This neuron was categorized as Pt_d_(LPOG)_ subtype^18,26^.

#### Bilateral transverse-tract neurons

The six other transverse-tract neurons were bilateral, forming two equally sized subtypes. One subtype, including three stained neurons, had dendritic arborizations in the LPOG of both ALs (Figs. 4f, 5c). This neuron subtype projecting to the contralateral PLP and LH, was previously reported in *M. sexta*^34^. We named it Pt_e_(LPOG)_. The second bilateral transverse-tract neuron subtype, comprising three neurons as well, originated in one LPOG only. The main axon passed ipsilaterally up to the ventrolateral edge of the CB. Here, it divided into two branches, one terminating in the LH and the VLP of the ipsilateral hemisphere and the other terminating in the VLP of the contralateral hemisphere (Figs. 4g, 5d). This neuron subtype, named Pt_f_(LPOG)_, is previously not described.

### LPOG neurons confined to the medial antennal-lobe tract bypass the calyces (Ca)

**Figure 6:**
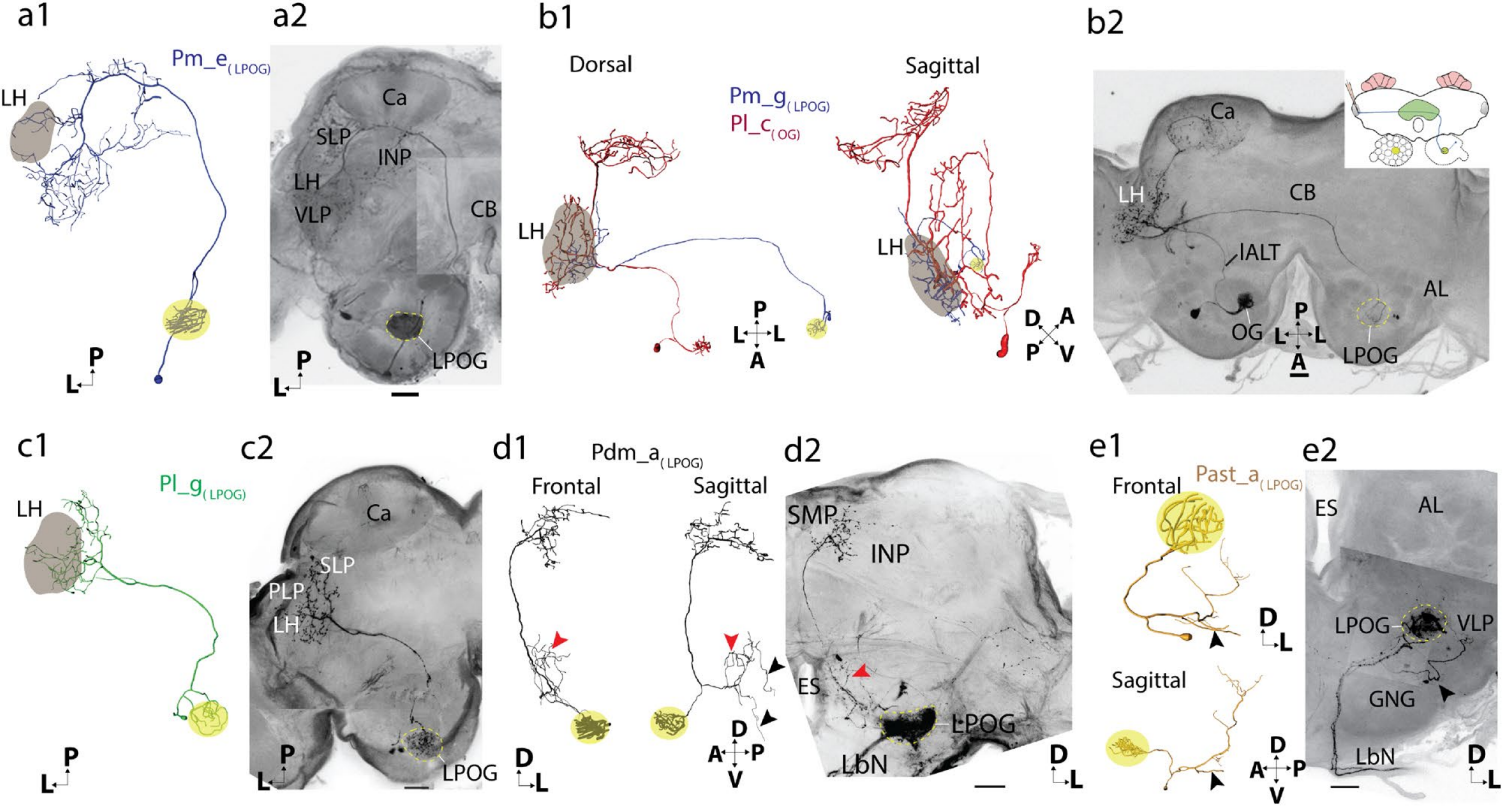
Morphologies of LPOG-neurons confined to the medial tract (**a-b**), lateral tract (**c**), dorsomedial tract (**d**) and antennal-subesophageal tract (**e**). (**a1, a2**) 3D reconstruction and confocal image of a medial-tract neuron of the Pm_e_(LPOG)_ subtype. The wide-spread terminals of this unilateral neuron targeted the lateral horn (LH), superior lateral protocerebrum (SLP), inferior neuropil (INP), and superior lateral protocerebrum (SLP). The axon of the neuron bypassed the calyces (Ca) on its anterior border. (**b1, b2)** 3D reconstruction and confocal image of a medial-tract neuron of the Pm_g_(LPOG)_ subtype (stained from the LH). The axon of this neuron followed the medial tract ipsilaterally before it turned, passed the brain midline, and terminated in the contralateral LH. This neuron was co-stained with a lateral-tract neuron from an ordinary glomerulus in the contralateral antennal lobe, showing the general overlap in the LH of output neurons from the LPOG and ordinary glomeruli. The schematic drawing in **b2** indicates the labeling site of the Pm_g_(LPOG)_ neuron, i.e., the contralateral LH. (**c1, c2**) 3D reconstruction and confocal image of a lateral-tract neuron of the Pl_g_(LPOG)_ subtype. This neuron projected to the lateral horn (LH) and superior lateral protocerebrum (SLP). Its cell body was localized in the lateral cell body cluster. **(d1, d2**) 3D reconstruction and confocal image of an LPOG neuron passing in the dorsomedial tract. Reconstruction of the Pdm_a_(LPOG)_ neuron showed the axon of the neuron dividing into two main branches, one dorsally projecting branch terminating in the superior medial protocerebrum (SMP) and the inferior neuropil (INP), and one ventrally projecting branch innervating the ventro-medial neuropil (VMNP, *red* arrowhead). The neuron also projected to the antennal mechanosensory and motor center (AMMC, *black* arrowhead) and the gnathal ganglion (GNG). (**e1, e2**) 3D reconstruction and confocal image of an LPOG neuron confined to the antennal-subesophageal tract (AST). The neuron, named Past_a_(LPOG)_, extended blebby terminals in the AMMC (*black* arrowhead). The cell body was localized in the GNG. Several fibers in the labial pit nerve (LbN), which project from the labial pit organ, were co-stained. (The reconstructions of dendrites in **a1, d1,** and **e1** are shown for illustrative purposes only. Due to prestaining of the LPOG, the detailed arborization pattern was not visible.) AL, antennal lobe; Ca, calyx; CB, central body. D, dorsal; V, ventral; P, posterior; L, lateral; A, anterior; VLP, ventrolateral protocerebrum; Scale bars: 50 μm.

One of the notable features of the mass-stained LPOG PNs was the weak innervation of the Ca—even when multiple medial-tract neurons were labeled (Fig. 3a). The single-neuron staining experiments affirmed this feature. The two medial-tract LPOG neurons individually stained here, formed two subtypes—both bypassing the Ca (Figs. 4h, i and 6a, b).

One unilateral medial-tract neuron originating in the LPOG bypassed the Ca but innervated various areas of the protocerebrum including the LH, SLP, PLP, and VLP (Figs. 4h, 6a). In addition, the axon extended a few side-branches into the inferior neuropil (INP). We classified this subtype as Pm_e_(LPOG)_, in accordance with previously reported neurons displaying similar morphologies^27^. The other medial-tract neuron originating in the LPOG projected to the contralateral protocerebrum (Figs. 4i, 6b). The axon of this subtype passed along the initial part of the ipsilateral medial ALT, however, then it turned and passed along the posterior edge of the CB, crossing the brain midline and targeting the contralateral LH exclusively. This new subtype was named Pm_g_(LPOG)_.

### A lateral-tract neuron connecting the LPOG directly to the LH

One of the 15 labeled LPOG output neurons projected along the lateral ALT. This neuron targeted various regions in the ipsilateral protocerebrum including the LH, PLP, and a small part of the SLP (Figs. 4j, 6c). The lateral-tract LPOG neuron had dense dendritic arborization in the LPOG. This newly described neuron was classified as Pl_g_(LPOG)_.

### LPOG neurons confined to minor tracts

In addition to the three LPOG neuron types described above, including PNs confined to the transverse, medial, and lateral ALT, the single neuron labeling technique explored two rare types, which are not frequently stained. One of these LPOG neurons projected in the dorsomedial ALT (dmALT), which is a relatively thin fiber bundle passing more dorsally and medially than the prominent medial tract. Whereas previously described neurons confined to the dorsomedial tract are reported to project bilaterally^26^, the neuron identified here projects unilaterally. From the LPOG, the main axon passed along the dmALT to the SMP, INP, and the ventro-medial neuropil (VMNP, Figs. 4k, 6d). In addition, a few fibers projected to the antennal mechanosensory and motor center (AMMC) and the gnathal ganglion (GNG). So far, no PN subtypes confined to the dmALT have been reported. This newly described neuron was therefore classified as Pdm_a_(LPOG)_.

One LPOG output neuron projected in the antennal suboesophageal tract (AST; Figs. 4l, 6e). The small cell body of the neuron (approximately 4.5 μm in diameter) was localized outside the AL, in the dorsolateral cell-body rind in the ipsilateral GNG. The neuron projected ventrally, side by side with the sensory bundle from the LPO. Then it turned laterally and sent terminal projections into the ventral area of the AMMC and VLP. This newly described neuron was classified as Past_a_(LPOG)_.

## Discussion

Here, we present new anatomical data on the central pathway processing CO_2_ information in the noctuid moth, *H. armigera.* Totally, 15 individual AL PNs originating from the LPOG were stained. This morphologically heterogenous neuron assembly included nine subtypes, most of which were not previously described. A considerable amount of PNs, i.e. ten, passed along the up to now poorly described transverse ALT. The typical medial-tract neuron innervating the Ca before terminating in the LH, was not found. In order to estimate the real number of LPOG output neurons, we combined the results from the mass-staining and single-unit labeling experiments. The highest number of LPOG PNs observed in the anterogradely mass-stained preparations were five (Fig. 3a), including the three ipsilateral subtypes, Pt_a_(LPOG)_, Pm_e_(LPOG)_, and Pl_g_(LPOG)_. From the retrograde staining performed from the lateral protocerebrum, including nine stained somata linked to the LPOG in the contralateral AL (Fig. 3d), we can conclude that at least nine bilateral/contralateral PNs originate from the LPOG (possibly making up Pt_e_(LPOG),_ Pt_f_(LPOG)_, and/or Pm_g_(LPOG)_ subtypes). In addition, we stained three distinct PN subtypes not represented in any of the mass-stained preparations, i.e. Pt_d_(LPOG)_, Pdm_a_(LPOG)_, and Past_a_(LPOG)_ (Fig. 4e, k, l). Taken together, we assume there are at least 17 PNs originating from the LPOG. This number is comparable with the amount of CO_2_ output neurons from the V-glomerulus in *Drosophila*, in which 12 PNs were imaged by using the photoactivatable green fluorescent protein technique^36^.

Notably, 12 of the 15 LPOG neurons presented here had overlapping projection terminals in the LH, which constitutes a main output target for PNs originating from the numerous ordinary glomeruli (see introduction). The comprehensive data on AL output neurons recently obtained from *H. armigera*—including both plant odor and pheromone neurons^25,26^, constitutes an excellent base for exploring putative interactions between the CO_2_ pathway and these two olfactory sub-systems.

### The CO_2_ pathway and the sub-systems devoted to plant-odors and pheromones

In Lepidoptera, CO_2_ and plant odors are detected by sensory neurons localized on different organs. From here, the two categories of sensory neurons project to distinct sets of AL glomeruli, i.e., the LPOG and the ordinary glomeruli, respectively. As both stimulus inputs are relevant for detecting the quality of host plants (see introduction), it is likely that the two signal pathways interact in the central nervous system. Given that all LPOG PNs are uniglomerular, as those identified in this study, there seems to be a continuation of the segregated paths also at the second-order level, implying a system organized mainly according to a labeled-line principle^37^. On the other hand, the LPOG is not totally segregated from the other glomeruli but interconnected via numerous multiglomerular LNs innervating the AL globally. In a recent study of AL neurons in *H. armigera*, about 90% of the LNs were reported to arborize in the LPOG^26^. Even though a substantial proportion of these LNs had relatively weak innervations in the LPOG, this indicates that processing does occur across the glomerular categories. Like in other insects, the main proportion of LNs in heliothine moths is GABAergic^38^. Indeed, we can’t rule out the possibility that LPOG PNs could be influenced by excitatory LNs^39–42^ or by polysynaptic GABAergic inputs^43^. Anyway, considering the typical global pattern of LNs arborizing in the LPOG, we suggest that a modest degree of signal integration takes place across the sub-systems at this level.

The finding of *uniglomerular* LPOG neurons exclusively, forming a distinct output pathway for CO_2_ information, as presented here, is in accordance with the labeled-line principle characterizing the two previously well-described olfactory sub-systems devoted to plant odors and pheromones, respectively^18,20,25,26,35^. Unlike the pheromone sub-system, having its distinct output area in the lateral protocerebrum, the CO_2_ sub-system has axon terminals overlapping with the plant-odor PNs. As shown in Fig. 6b, reconstructions of two co-stained neurons, one from the LPOG and the other from an ordinary glomerulus, demonstrates the overlapping terminals in the LH. This overlap indicates that the LH is a significant region for integration of sensory inputs about plant odors and CO_2_. One putative downstream site for receiving information processed in the LH is the SMP^44^. Interestingly, the SMP is also directly innervated by at least two LPOG neuron subtypes identified here (Pt_d_(LPOG)_ and Pdm_a_(LPOG)_). Thus, to record response-patterns of SMP-neurons during stimulation with plant-odors and CO_2_ constitutes an exciting subject for future studies. Taken together, we suggest that the LH is an essential site for integrating CO_2_ and plant odor signals.

In contrast to the substantial overlap with parts of the plant odor sub-system, the LPOG projections had weak innervations in the main target areas of the male-specific MGC neurons, including the SLP and the superior intermediate protocerebrum (SIP)^25^. Although some of the LPOG PNs stained here, i.e. four neuron subtypes (Pt_a_(LPOG)_, Pt_d_(LPOG)_, Pm_e_(LPOG)_, and Pl_g_(LPOG)_), had axonal terminals in the SLP, they targeted a distinct area localized more ventrally and lateral-posteriorly as compared to the MGC PNs^25,35,45^.

The finding that PNs carrying CO_2_ signals interact more intensively with plant odor PNs than with pheromone PNs fits well with previous studies in *Drosophila* demonstrating extensive synaptic interactions in the LH between cholinergic PNs^46^. These cholinergic PNs include neurons responding to CO_2_^47,48^. In addition, a population of plant/food odor PNs also target the same region^49^. Overall, the results presented here take us one step further towards understanding the neuronal architecture underlying moths’ ability to measure fine fluctuations in CO_2_ for determining the nutritional quality of relevant host plants^5,50^.

### CO_2_ signaling is modestly involved in sensory learning

The calyces of the mushroom bodies are a neuropil for sensory integration and memory, important for experience-dependent reactions^51–55^. In contrast to the main olfactory pathway, which innervates the Ca extensively (Fig. 1), the LPOG PNs had a rather restricted innervation of this neuropil. Both the mass-stained and single-unit stained preparations demonstrated considerably less terminals in the Ca than in the LH (Figs. 3a, 5,6). Actually, only one of the nine LPOG neuron subtypes, Pt_a_(LPOG)_, innervated the Ca. Its distributed and blebby terminals in the Ca correspond with the features of the typical uniglomerular plant-odor PNs and differ from the innervation pattern of pheromone PNs^18,25,35^. In the fruit fly, PNs tuned to CO_2_, which are connected with the V-glomerulus, also innervate the Ca modestly; the only neuron type targeting this neuropil is a bilateral lateral-tract neuron^36^. The general projection pattern of LPOG neurons bypassing the Ca suggests that the CO_2_ signaling system is minimally involved in sensory learning, particularly olfactory learning.

The apparent trivial role of CO_2_ input for memory suits well with the fact that this gas is ubiquitous, serving as a minor compartment of the Earth’s atmosphere. Like the CO_2_ PNs, other AL output neurons tuned to universal cues, such as temperature and humidity, also display little or no connection with Ca. For instance, both in the fruit fly and cockroach, PNs carrying thermo- and hygro-sensory signals have modest or no innervation in the Ca^32,56^.

### Functional implications of CO_2_ signaling in the other brain areas

In addition to targeting the LH, the CO_2_ PNs identified here also projected to various other neuropils, such as VLP, PLP, and SMP (see Fig. 4 and Table 1). In fruit flies, output neurons from the V-glomerulus, which corresponds with the LPOG, are reported to terminate in similar neuropil regions^36^. One major difference between the CO_2_ systems in moth and fruit fly is that seemingly all LPOG neurons in moth are uniglomerular whereas the majority of those connected with the V-glomerulus in the fly is multiglomerular. Whether this is related to the peripheral organization in flies including CO_2_ sensory neurons co-located with olfactory neurons on the antenna, is an open question. Regardless of dendritic arrangement in the AL, however, the second-order pathways for CO_2_ signaling in the two insect groups are rather comparable.

Given these similarities across two insect model species, it is encouraging to speculate about the functional roles of these neuropils. The VLP, which is one of the major innervation areas of olfactory PNs^27^, is also targeted by neurons carrying information about vision^57^, audition^58^, and temperature and humidity^32^. We found that the VLP region is targeted by two transverse-tract LPOG subtype neurons, Pt_a_(LPOG)_ and Pt_f_(LPOG)_, and an antennal subesophageal tract neuron, Past_a_(LPOG)_. Thus, the data presented here, coincides with the previous studies demonstrating the involvement of the VLP in integration of multimodal information. The PLP, which also constitutes an essential target area for olfactory neurons^27^, is reported to receive visual information both in fruit flies^32,57^ and in the silk moth^59^. In the data presented here, we found at least four LPOG neuron subtypes (Pt_a_(LPOG)_, Pt_e_(LPOG)_, Pm_e_(LPOG)_, and Pl_g_(LPOG)_) targeting the PLP region. Taken together, the previously considered integration areas for multimodal processing, the VLP and PLP, receive input about CO_2_ as well.

### Diverse transverse-tract neurons indicate their multifunctional roles

In *H. armigera*, the transverse-tract neurons originate frequently from glomeruli localized in the ventral and posterior part of the AL^26^. We noticed that the majority of stained LPOG neurons, i.e., 10 of 15, were confined to this ALT. They are further classified into four morphological subtypes. Two subtypes, Pt_a_(LPOG)_ and Pt_e_(LPOG)_, were previously described in *M. sexta*^34^—by then, they were classified as an inner-tract PIa(G) neuron and a protocerebral neuron, respectively. Unlike the classic ALTs (including the medial, lateral, and mediolateral ALT), the transverse ALT is known to be formed by a relatively restricted number of neurons displaying diverse morphologies^27,28^. This implies that the target areas of the transverse-tract neurons are less confined as compared to neurons in other tracts. A previous study on the fruit fly reported transverse-tract neurons originating from two posteriorly located glomeruli conveying information about temperature and humidity^32^. Notably, these non-odor responding neurons in the fly are morphologically comparable to some transverse-tract PNs found in *H. armigera*. Generally, the restricted number of neurons confined to the transverse ALT and their high degree of heterogeneity indicate that, in contrast to the main tracts, the tALT is formed by a few functionally unique PNs.

## Conclusion

The anatomical data presented here, including visualization of individually labeled LPOG PNs forming the second-order pathway for CO_2_ signals in the moth brain, contribute to improve our general understanding of parallel olfactory systems in insects. Besides, the high-resolution confocal data of individual neurons and neuron populations form the basis for future experiments exploring both anatomical and physiological characteristics of the central pathways involved in processing input about external fluctuations in CO_2_.

## Materials and methods

### Insects and preparation

Male and female *Helicoverpa armigera* pupae (Lepidoptera; Noctuidae, Heliothinae), obtained from Keyun Bio-pesticides (Henan, China), were allowed to eclose in climate chambers (Refritherm 200 and 6E, Struers-Kebolab, Albertsund, Denmark, or Binder KBF 720, Tuttlingen, Germany) at 24 °C and 70% air humidity on a 14:10 h light/dark cycle (lights on at 18:00). After emergence, the moths were supplied a 10% sucrose solution. The moths were 1–4 days old when the experiments were performed. According to Norwegian law of animal welfare, there are no restrictions regarding experimental use of Lepidoptera.

Preparation of the insect has been described in detail elsewhere^13,25^. Briefly, the moth was restrained inside a plastic tube with the head exposed and then immobilized with dental wax (Kerr Corporation, Romulus, MI, USA). The brain was exposed by opening the head capsule and removing the intracranial muscles and tracheas. The exposed brain was continuously supplied with Ringer's solution (in mM: 150 NaCl, 3 CaCl_2_, 3 KCl, 25 sucrose, and 10 N-tris (hydroxymethyl)-methyl-2-amino-ethanesulfonic acid, pH 6.9).

### Iontophoretic staining of individual PNs originating from the LPOG

The procedure of iontophoretic staining of AL PNs was performed as previously described^25,27,35^. Sharp electrodes were made by pulling quartz capillaries (OD: 1 mm, ID: 0.5 mm, with filament; Sutter instrument) on a horizontal puller (P-2000, Sutter instruments, CA, United States). The tip of the electrode was filled either with 4% biotinylated dextran-conjugated tetramethylrhodamine (3000 mw, micro-Ruby, Molecular Probes) in 0.2 M potassium acetate (KAc) or 4% Alexa Fluor 488 dextran (10,000 mw, Molecular Probes) in distilled water. A chloridized silver wire placed in the eye served as a reference electrode. The recording electrode having a resistance of 200–300 MΩ, was backfilled with 0.2 M KAc. Iontophoretic staining of single neurons was conducted in two ways. (i) We carefully inserted the electrode into the LPOG region from the dorsal AL area (n = 10) via a micromanipulator (Leica, Wetzlar, Germany). Contact with an LPOG-neuron was confirmed by a response when applying a CO_2_ puff (Fig. 3a). (ii) We pre-stained the LPO sensory neurons with micro-Ruby 48 h prior to the iontophoretic staining of LPOG output neurons. With clear visualization of the LPOG under a Zeiss stereo discovery V12 microscope equipped with epifluorescence, we inserted the electrode into the LPOG from a frontal position (n = 5). Each neuron was stained by applying 200 ms depolarizing current pulses of 2–3 nA, at 1 Hz, for 10 min. After labeling, the insects were kept overnight at 4 °C in dark to allow anterograde axonal transportation of the dye. Then the brain was dissected from the head capsule and fixed in a paraformaldehyde solution (4% PFA in 0.1 M phosphate buffer, pH 6.9) for 2 h at room temperature or overnight at 4 °C before it was dehydrated in an ascending ethanol series (50%, 70%, 90%, 96%, and 2 × 100%; 10 min each), and finally cleared in methyl salicylate (Sigma- Aldrich, Germany).

### Mass staining of output neurons originating from the LPOG

Mass staining of LPOG projection neurons was conducted in 8 male moths. Here, we applied the fluorescent dye into the relevant AL region via a sharp electrode with low resistance (~ 40 MΩ). Borosilicate capillaries (OD: 1 mm, ID: 0.5 mm, with filament 0.13 mm; Hilgenberg GmbH, Germany) were pulled on a horizontal puller (P-97, Sutter instruments, CA, United States). The tip of the glass electrode was filled with 4% micro-Ruby solution. The staining site was identified by testing the local field potentials during CO_2_ stimulation (Fig. 3a). After that, the region was mass stained by applying a series of depolarizing current pulses of 50 nA at 1 Hz for 30 min. In addition to the anterograde mass staining, two types of retrograde labeling experiments were performed including application of dye crystals into the Ca and the LH, respectively. The dissection and dehydration were conducted as described above.

### Stimulation delivery

During the electrophysiological recording, a pulse of CO_2_ was delivered by a stimulation system including two parallel paths, one carrying a continuous airstream and the other the 400 ms CO_2_ stimulus (100%, AGA AS, Oslo, Norway). A solenoid valve system (General Valve Corp.) regulated the application of the stimulus. To identify the neuron responses, application of the CO_2_ stimulus and the control (fresh air) was repeated three times.

### Confocal microscopy

Brains with successfully stained neurons were imaged using a confocal laser scanning microscope (LSM 800, Zeiss, Jena, Germany), equipped with C-Apochromat 10x/0.45 water objective, C-Apochromat 10x/0.3 air objective, and Plan-Neofluar 20x/0.5 air objective. Brains stained with micro-Ruby were scanned with a HeNe laser at 561 nm, and the emitted light was filtered through a 560–600 nm band pass filter. Preparations stained with Alexa Flour 488 were scanned with an Argon laser at 493 nm, including a 505–550 nm band pass filter. A synapsin-labeled preparation was carefully scanned utilizing two channels, in order to show that the neuropil structures visualized by the auto-fluorescent signal of endogenous fluorophores was in great agreement with the synapsin signal (Supplementary, Fig. S3). Thus, relevant structures in the brain containing the stained neurons were visualized by imaging the auto-fluorescence. Since many auto-fluorescent molecules in the tissue are excited at 493 nm, an Argon laser at 493 nm in combination with a 505–550 nm band pass filter was used. For all confocal scans, serial optical sections with a resolution of 1024 × 1024 pixels were obtained at 1.5–9 µm intervals through the entire depth of brain. The confocal images shown in this study were edited in ZEN 2.3 (blue edition, Carl Zeiss Microscopy GmbH, Jena, Germany). Brightness and contrast of the images were adjusted in Photoshop, version 21.

### Reconstruction and registration of neurons into the representative brain

The 3D reconstruction of relevant neuropil structures was based on the previously conducted confocal stacks for the representative brain^25^. We used the nomenclature established by Ito, et al.^60^ with the exceptions of the VLP. Our definition of the VLP, included the anterior and posterior VLP, along with the wedge. Furthermore, the superior intermediate protocerebrum (SIP) located adjacent to the SMP, was not specifically indicated in this study since none of the identified neurons projected to the SIP.

Individually labeled neurons and the surrounding brain neuropils were manually reconstructed from consecutive confocal sections by means of the visualization software AMIRA 5.3; the neurons were reconstructed by using the skeleton module of the software^61,62^ and the brain structures by using the segmentation editor. Thus, each neuron was traced so that a surface model built by cylinders of distinct lengths and thicknesses was created. The relevant neuropils were reconstructed based on the autofluorescence signals, as described above. To compensate for the refraction indexes, the z-axis dimension of the neuron and brain structures was multiplied by a factor of 1.16 for the water lens objective and 1.54 for the dry lens. Manual registration of individual neurons into a representative brain from Chu et al.^25^ followed the same procedure as described in Ian et al.^27^.

To provide a framework for determining the target areas of the LPOG PNs, we reconstructed nine new protocerebral regions from the confocal image stack previously utilized for creating the representative brain model^25^, utilizing the segmentation editor of AMIRA. Unspecified neuropil regions have previously been characterized in the lepidopteran brain by Ian et al.^27^ and Heinze and Reppert^63^.

### Image intensity processing and statistical analysis

As retrograded staining from the Ca labeled dendrites of all AL PNs linked with this neuropil, we could investigate putative differences in connection pattern between the LPOG and a typical ordinary glomerulus by measuring the glomerular fluorescence intensities. Here, we compared the fluorescence intensities in the LPOG and the neighboring glomerulus, G71, in six moths. We selected four confocal sections including all parts of each glomerulus. The fluorescence intensity within each of these two glomeruli was quantified by using Image J, respectively (https://imagej.net). Paired sample *t* test was performed to compare the mean intensities. All probabilities given are two-tailed. The Statistical package for the social sciences (SPSS), version 25, was used for statistical analysis.

## Supplementary information

Supplementary file 1
