# Supplementary material for: Neuronal architecture of the second-order CO2 pathway in the brain of a noctuid moth
Source: Sci Rep. 2020 Nov 16;10:19838. doi: 10.1038/s41598-020-76918-1 (PMC7669840; doi:10.1038/s41598-020-76918-1)
Supplement: Supplementary file 1 [file 41598_2020_76918_MOESM1_ESM.docx]

**Supplementary file for**

**Neuronal architecture of the second-order CO_2_ pathway in the brain of a noctuid moth**

Chu X^1+^, KC P^1+^, Ian E^1^, Kvello P^2^, Liu Y^3^, Wang GR^3^, Berg BG^1*^

^1^Chemosensory laboratory, Dept. of Psychology, Norwegian University of Science and Technology, NTNU

^2^Department of Teacher Education, Norwegian University of Science and Technology, NTNU

^3^State Key Laboratory for Biology of Plant Diseases and Insect Pests, Institute of Plant Protection, Chinese Academy of Agricultural Sciences, Beijing, China

^+^These authors contributed equally to this work.

*Correspondence and requests for materials should be addressed to BG Berg (e-mail: [bente.berg@ntnu.no](mailto:bente.berg@ntnu.no))

This file includes:

Supplementary Figures S1 to S3

***Supplementary Figure S1***

Mass-stained preparation shown in Figure 3a. The confocal images from two different depths of the frontally oriented brain illustrate that the mass filling site was localized to the LPOG. As shown, this glomerulus is situated most ventrally in the antennal lobe (AL), adjacent to the lateral cell body cluster (LC). The labeling of the labial pit nerve (LbN) and the contralateral LPOG also confirm that the staining site is the LPOG. GNG, gnathal ganglion. D, dorsal; V, ventral; L, lateral; Scale bars: 50 μm.

***Supplementary Figure S2***

Comparison of fluorescence intensities between the LPOG and the neighboring glomerulus, G71, in six preparations retrogradely stained from the calyces. (**a**) Quantification of the mean fluorescence intensity of LPOG and G71, respectively (n = 6). (**b**) Confocal image of one antennal lobe, showing the LPOG and G71 in dorsal view. Scale bar: 50 μm.

***Supplementary Figure S3***

Dual-channel confocal scanning showing the consistency between the anti-synapsin labeling and the autofluorescence signal. Scale bars: 50 μm.

***Supplementary Figure S1***

***
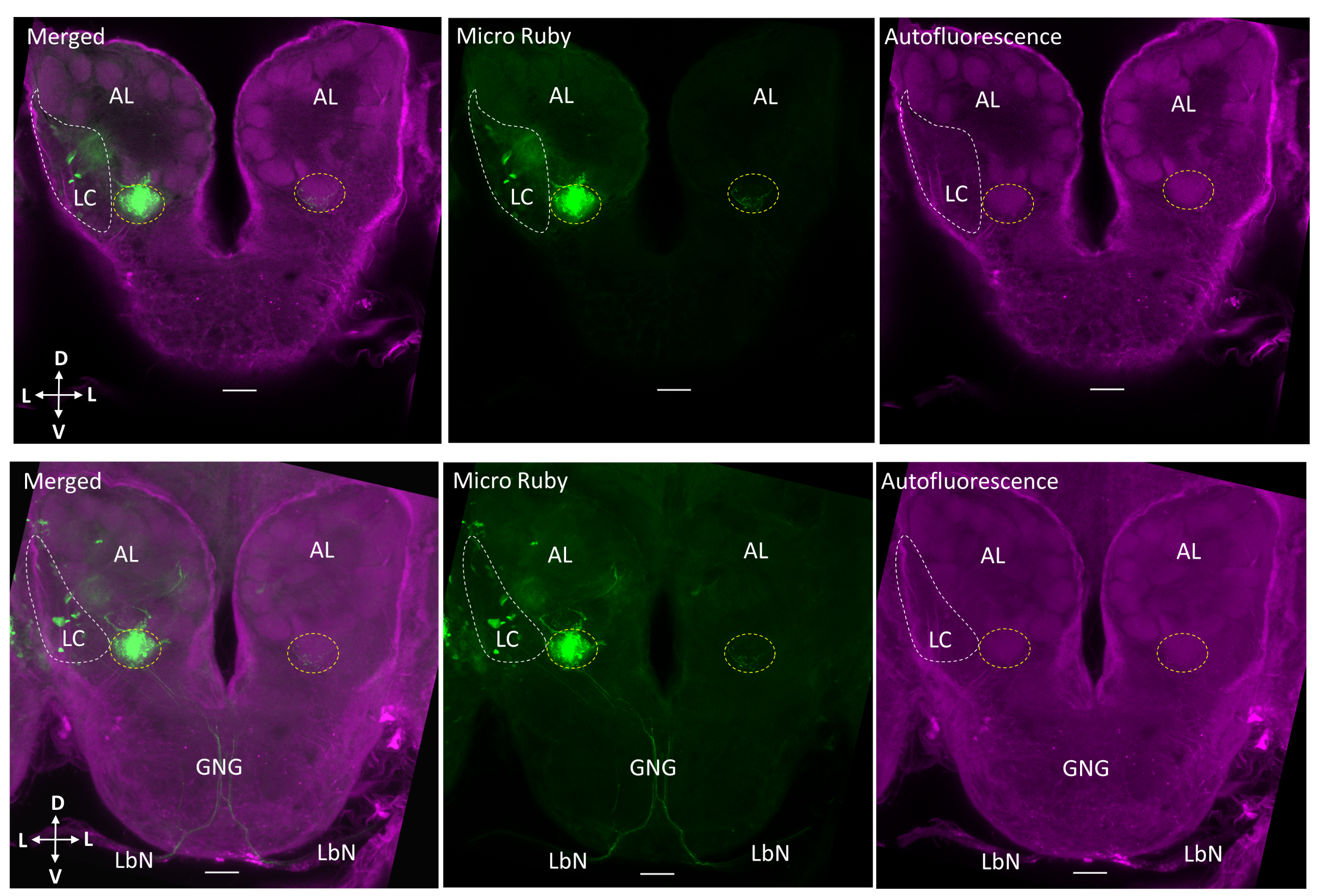
***

***Supplementary Figure S2***

***
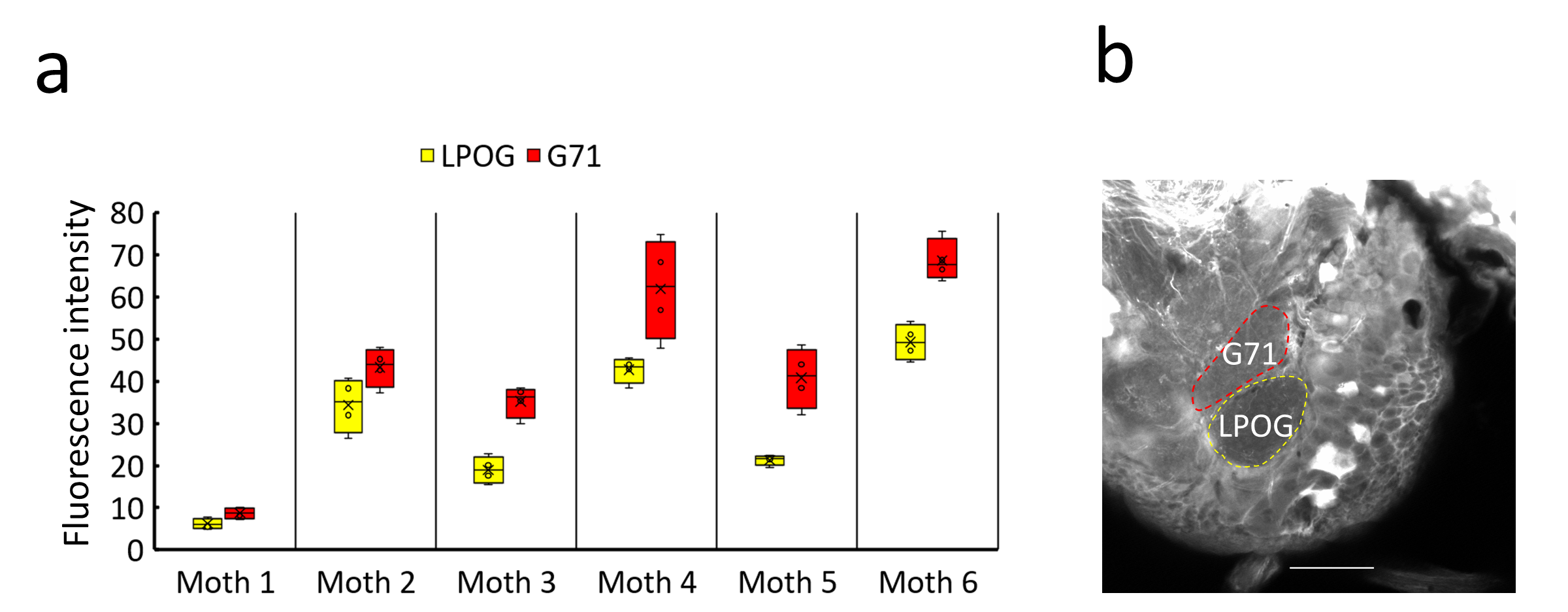
***

***Supplementary Figure S3***

***
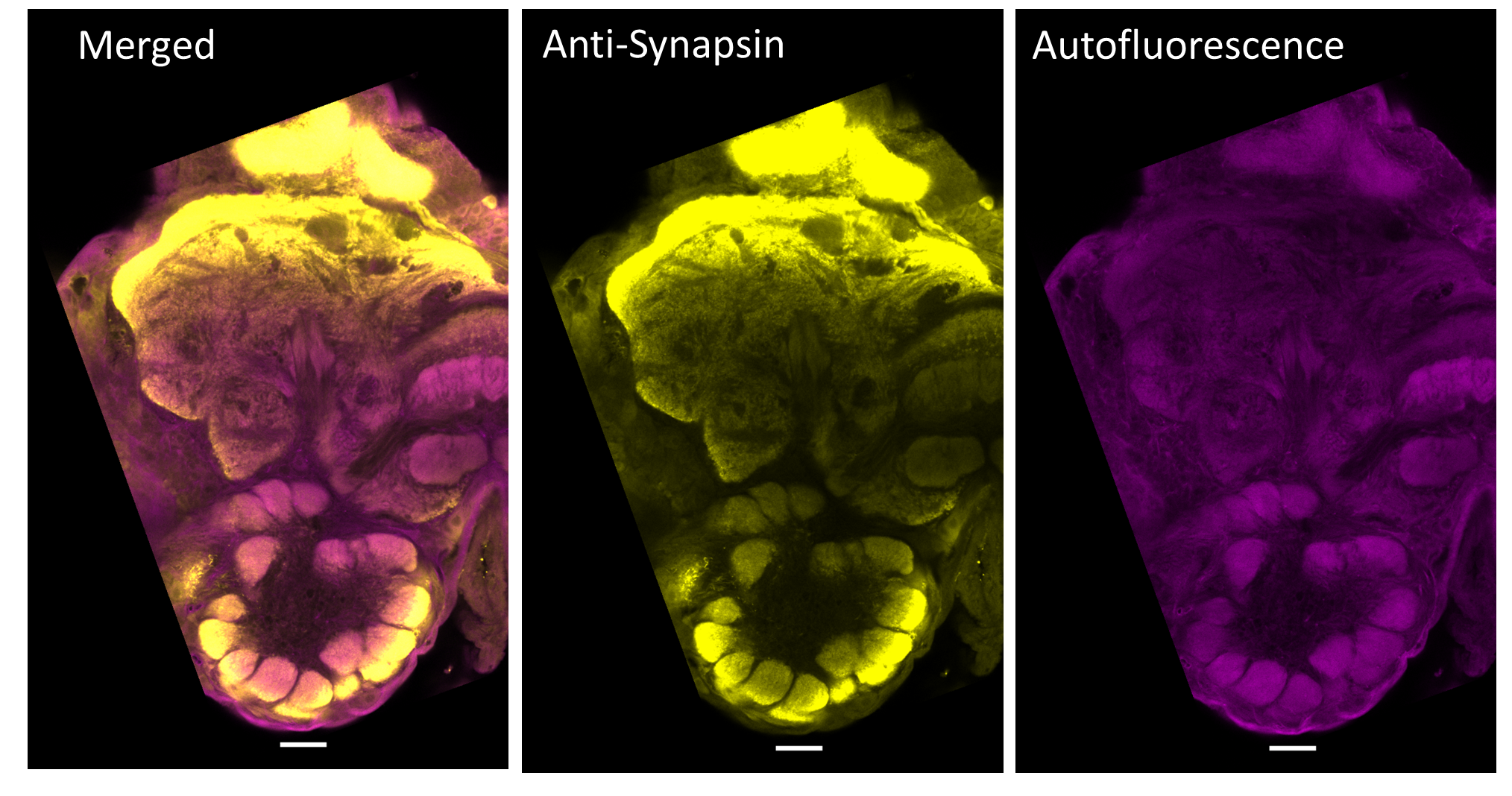
***
